# Supplementary material for: Host Dynamics under General-Purpose Force Fields
Source: Molecules. 2023 Aug 8;28(16):5940. doi: 10.3390/molecules28165940 (PMC10458655; doi:10.3390/molecules28165940)
Supplement: Supplementary file 1 [file molecules-28-05940-s001.zip › molecules-2526586-supplementary.pdf]

Supporting Information:

Host Dynamics under General-Purpose Force Fields

Xiaohui Wang [1,2], Zhe Huai [3] and Zhaoxi Sun [2,*]

[1] Beijing Leto Laboratories Co., Ltd., Beijing 100083, China
[2] College of Chemistry and Molecular Engineering, Peking University, Beijing 100871, China
[3] XtalPi—AI Research Center, 7F, Tower A, Dongsheng Building, No. 8, Zhongguancun East Road, Beijing 100083, China
[*] Correspondence: z.sun@pku.edu.cn

Figure S1. Deviations of force-field energetics from the B97-3c reference for the CB7 host: a) the transferable GAFF and the refitted FM-B97-3c set restrained to the GAFF initial guess and b) GAFF2 and the refitted FM-B97-3c-from-GAFF2 force fields. c-f) The corresponding time series of atom-specific force errors. In numbering of the host atoms, we purposely separate different elements. The first 28 atoms are nitrogen atoms in the backbone of the 7 repeating units (i.e., 4 N atoms in each unit), the last 14 atoms are oxygen in the -C=O portals (2 O atoms in each unit), and the middle part includes all carbon and hydrogen atoms. The hydrogen atoms do not have noticeable problems (white dots) and are thus mixed with carbon atoms in numbering. The assessment configurations and reference data independent from the fitting/training set are generated with the refitted force field FM-B97-3c for each initial guess. We can see that the largest contributions to the overall force errors are from the backbone N and C atoms, while the O atoms in the -C=O portals have relatively small force errors. Force-field refitting could effectively decrease the force errors of all heavy atoms to similar levels.

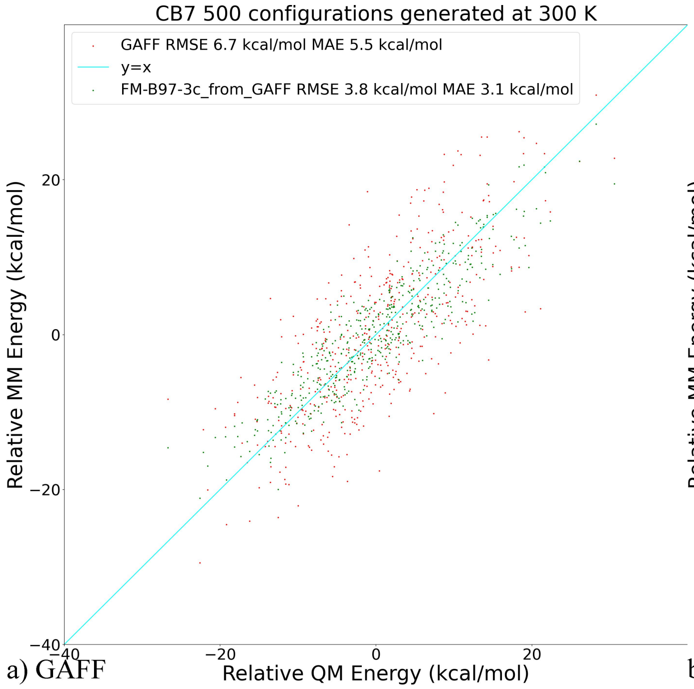

a) GAFF

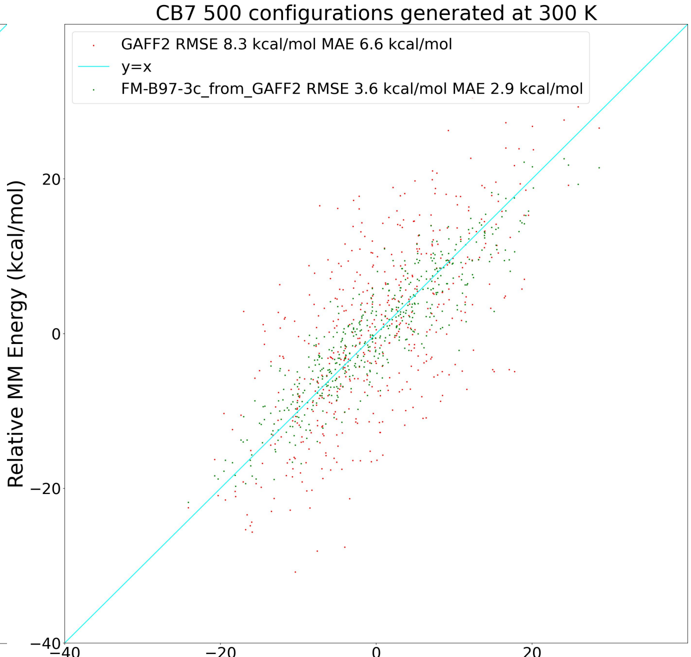

b) GAFF2

>30 kcal/(mol·Å) >20 kcal/(mol·Å) >10 kcal/(mol·Å)

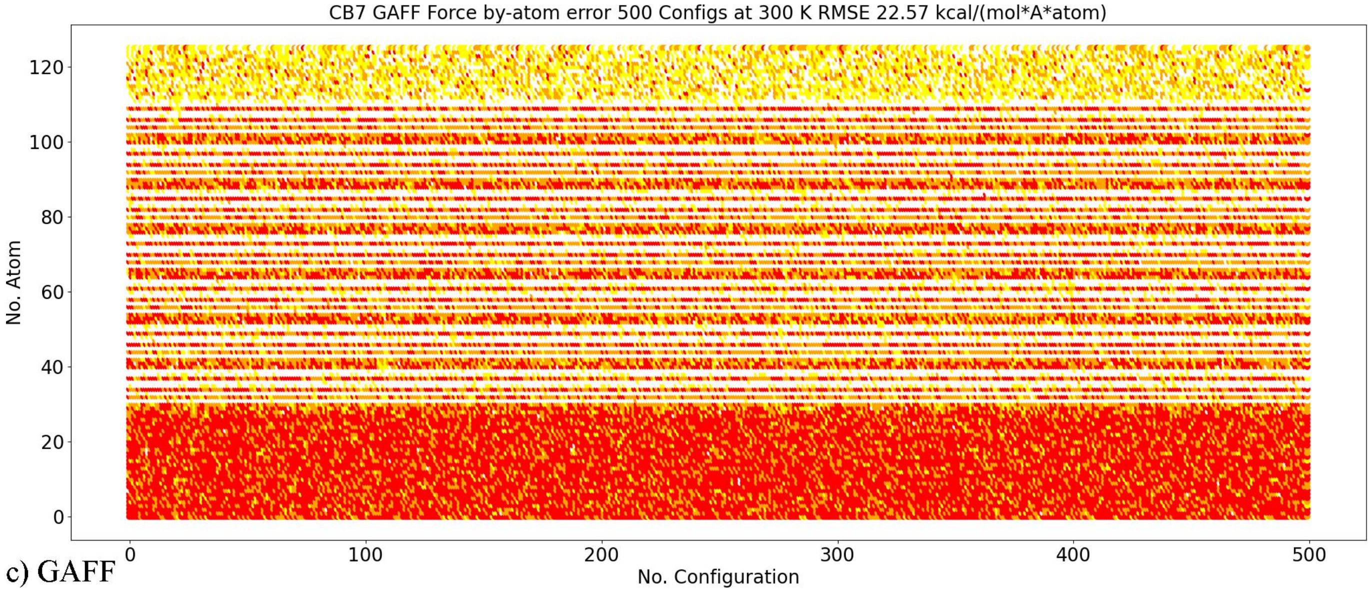

c) GAFF

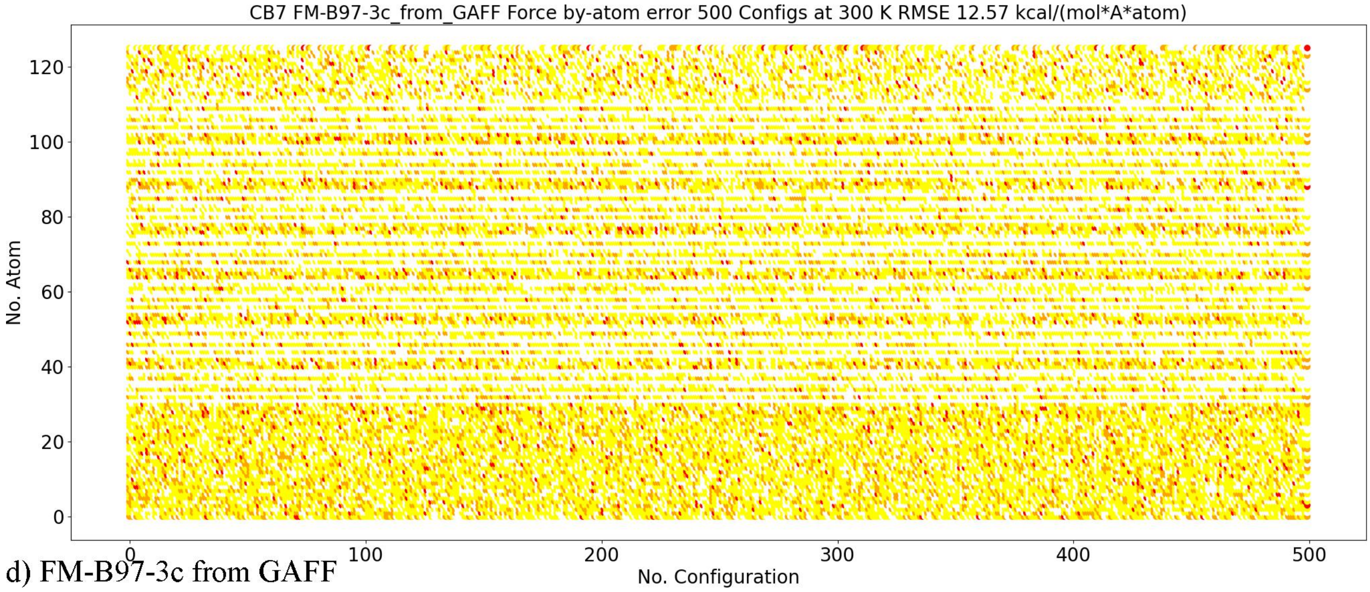

d) FM-B97-3c from GAFF

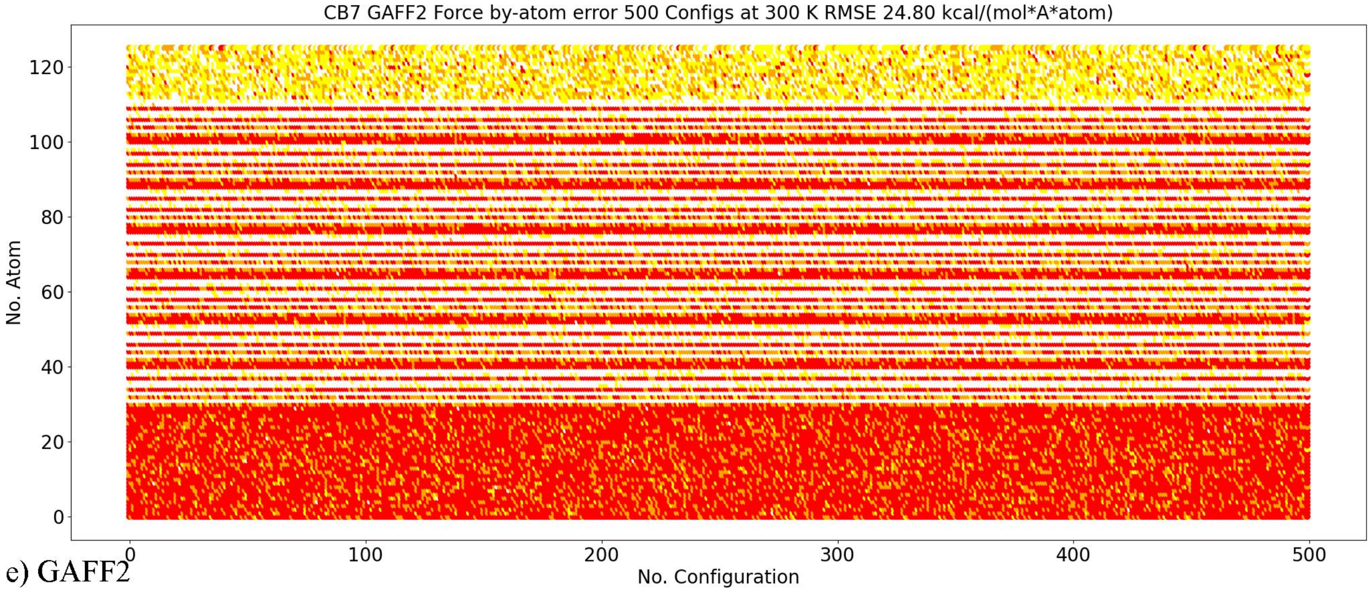

e) GAFF2

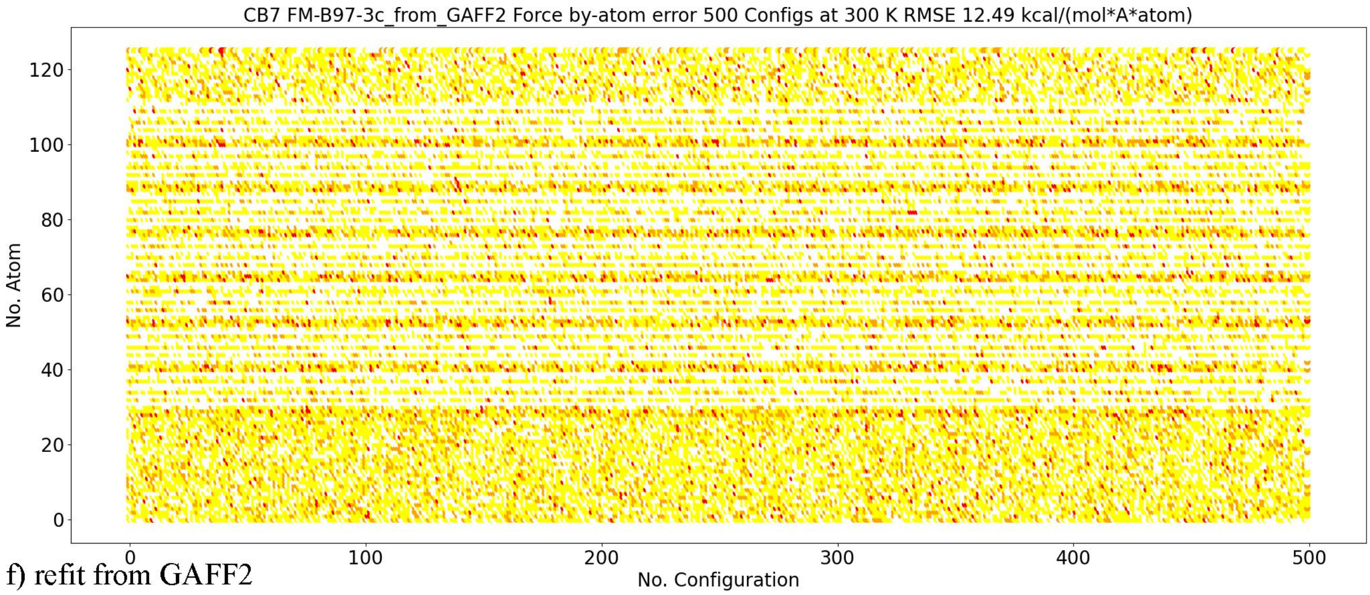

f) refit from GAFF2

Figure S2. CB7 host dynamics produced by refitted force fields: superpositions (5 ns per snapshot) of the host structure during the 500 ns explicit-solvent sampling of the solvated host under FM-B97-3c with either a) GAFF or b) GAFF2 initial guess, and c) Time series of radius of gyration (Rg) of the host molecule (sampling interval 50 ps). The refitted FM-B97-3c parameter sets, regardless of the initial guess, behave similarly to the pre-fitted GAFF and GAFF2 force fields in both the structure overlay and the radius of gyration, which suggest that both pre-fitted force fields are good enough for the current CB[*n*] host with 7 repeating unit. However, for enlarged rings such as CB8, the refitted and pre-fitted force fields behave somehow differently due to the increased structural flexibility, as observed in our previous work.

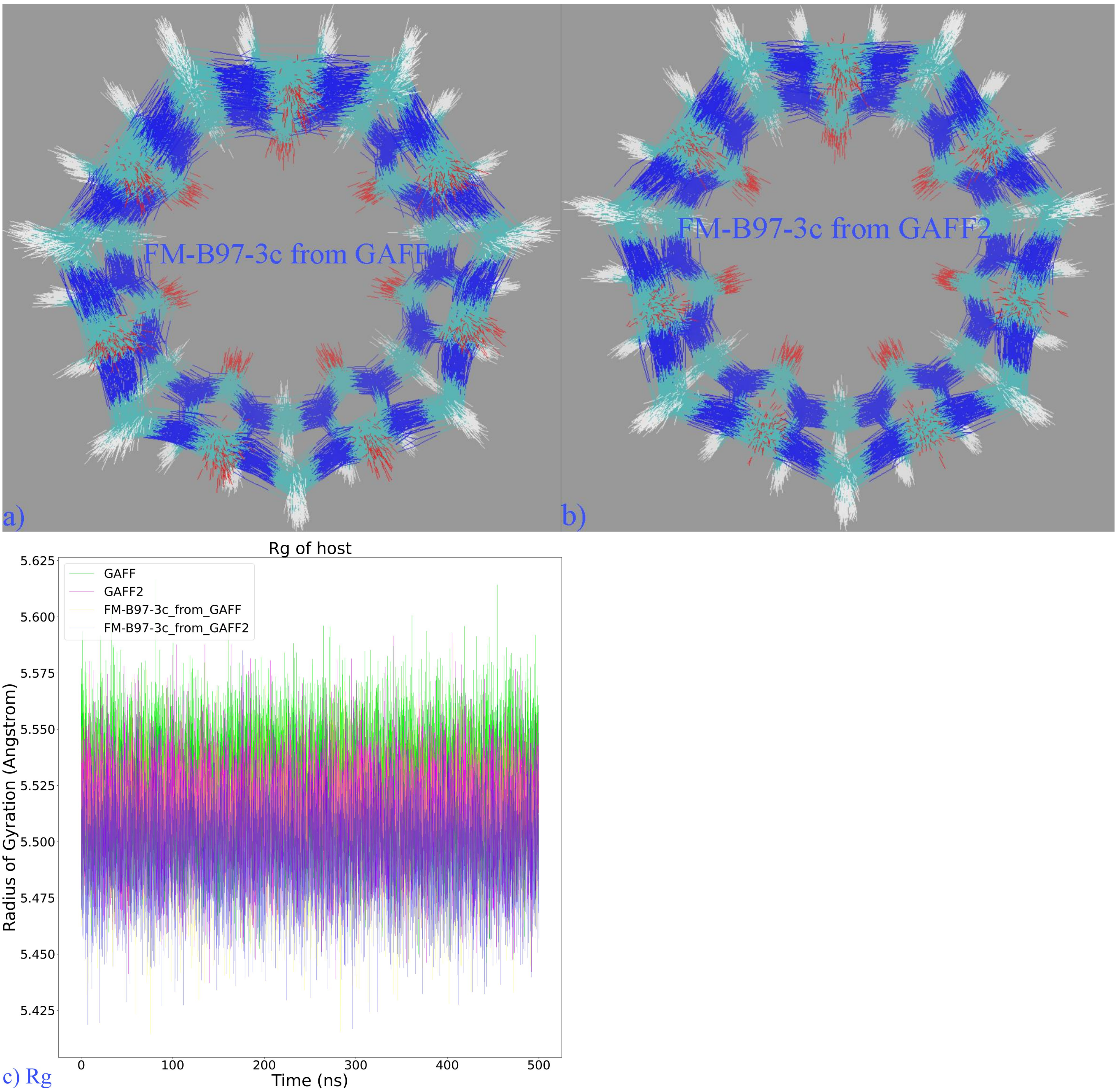

Figure S3. Deviations of force-field energetics from the B97-3c reference for the SP6 host: a) the transferable GAFF and the refitted FM-B97-3c restrained to the GAFF initial guess and b) GAFF2 and the refitted FM-B97-3c-from-GAFF2 force fields. c-f) The corresponding time series of atom-specific force errors. The 6 repeating units are numbered sequentially in the by-atom force errors, with the first 11 atoms being the pillararene backbone and the 12-21 atoms being the two -OSO_3^- rims. In the inset of the subplot a), the atom numbering in each repeating unit is presented. The assessment configurations and reference data independent from the fitting/training set are generated with the refitted force field FM-B97-3c for each initial guess. We can see that the force-field refitting leads to pronounced betterments in both energetics and atomic forces, and the improvements happen mostly on the -SO_4^- rims. By further comparing the term-specific parameters before and after refitting, we identify that the bond stretching term S-O marked in Fig. 5c is the most problematic point in both GAFF derivatives and is the origin of significant betterments upon refitting. Both its equilibrium length and force constant are altered significantly, e.g., bond length varying by ~7%.

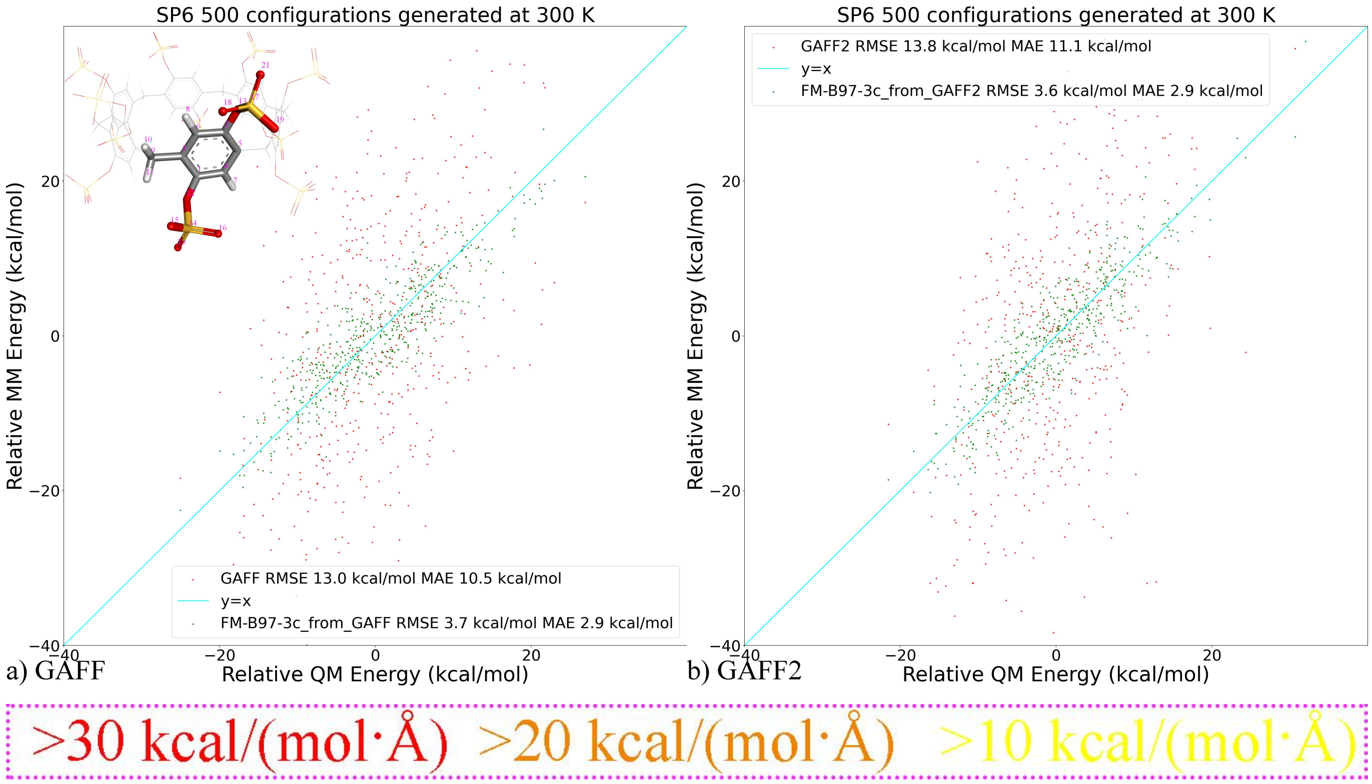

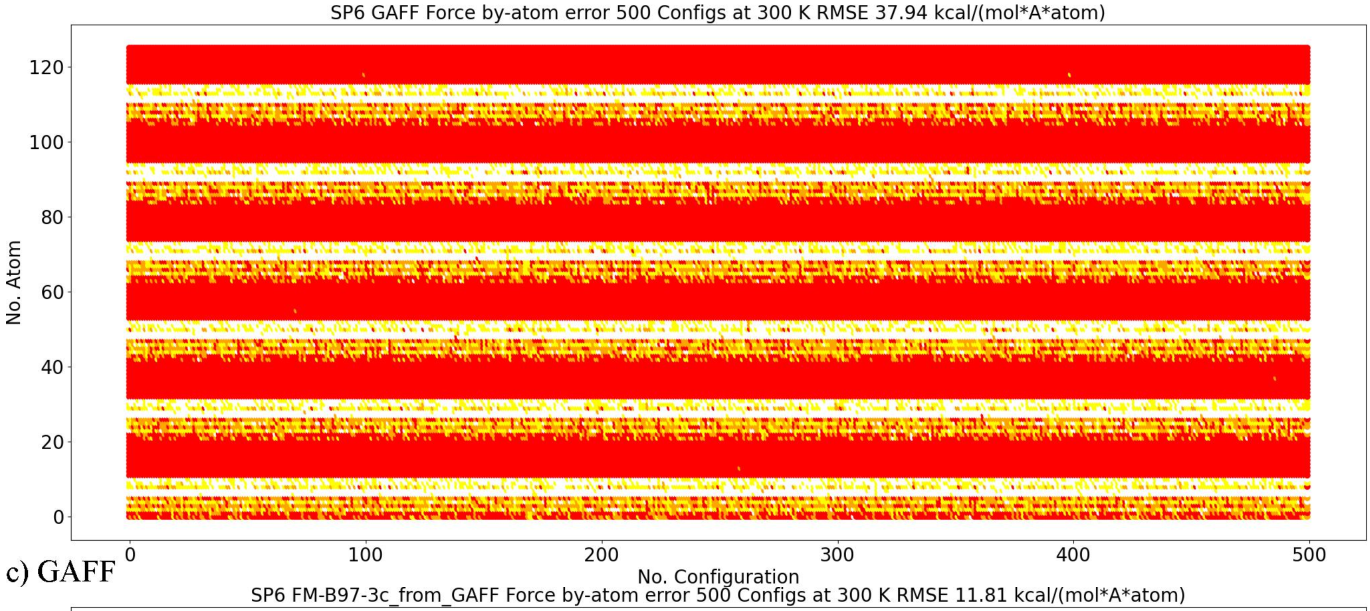

c) GAFF

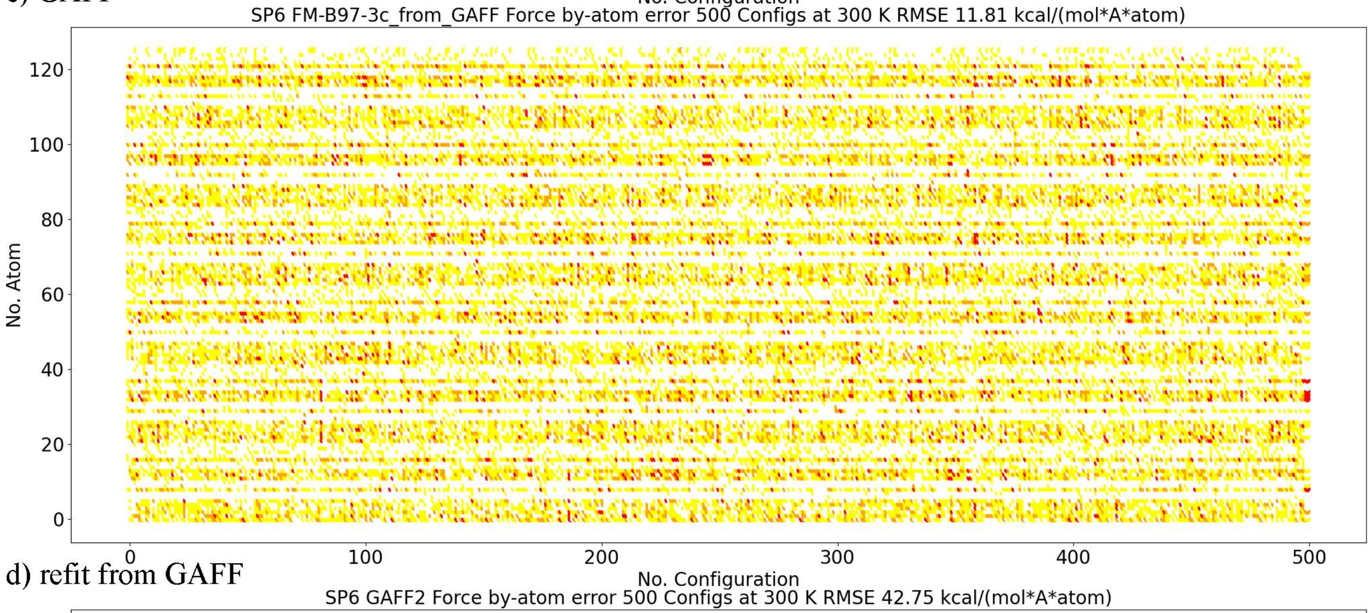

d) refit from GAFF

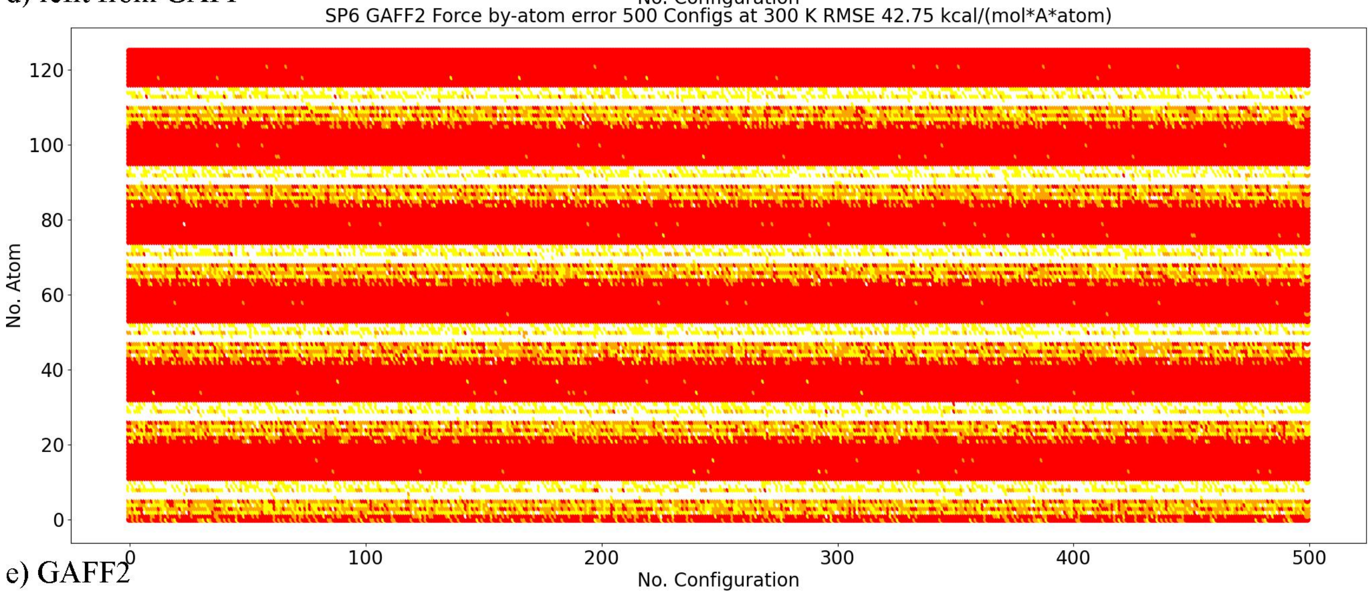

e) GAFF2

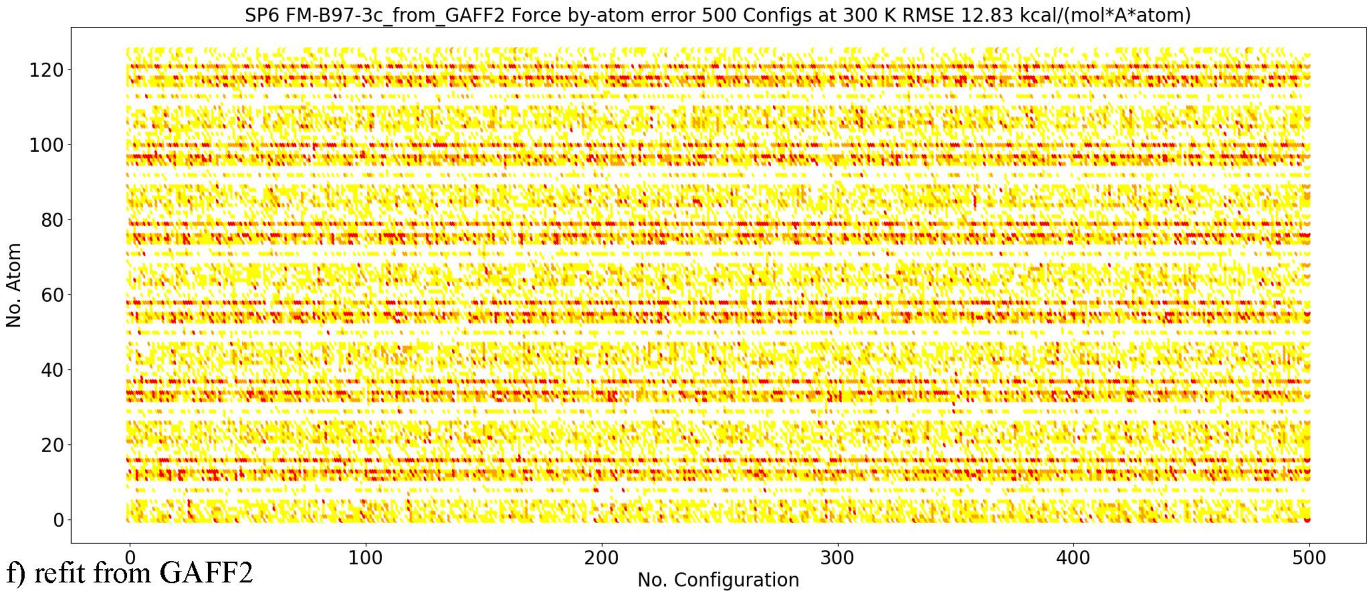

f) refit from GAFF2

Figure S4. SP6 host dynamics produced by refitted force fields: superpositions (5 ns per snapshot) of the host structure during the 500 ns explicit-solvent sampling of the solvated host under FM-B97-3c with either a) GAFF or b) GAFF2 initial guess, and c) Time series of radius of gyration (Rg) of the host molecule (sampling interval 50 ps).

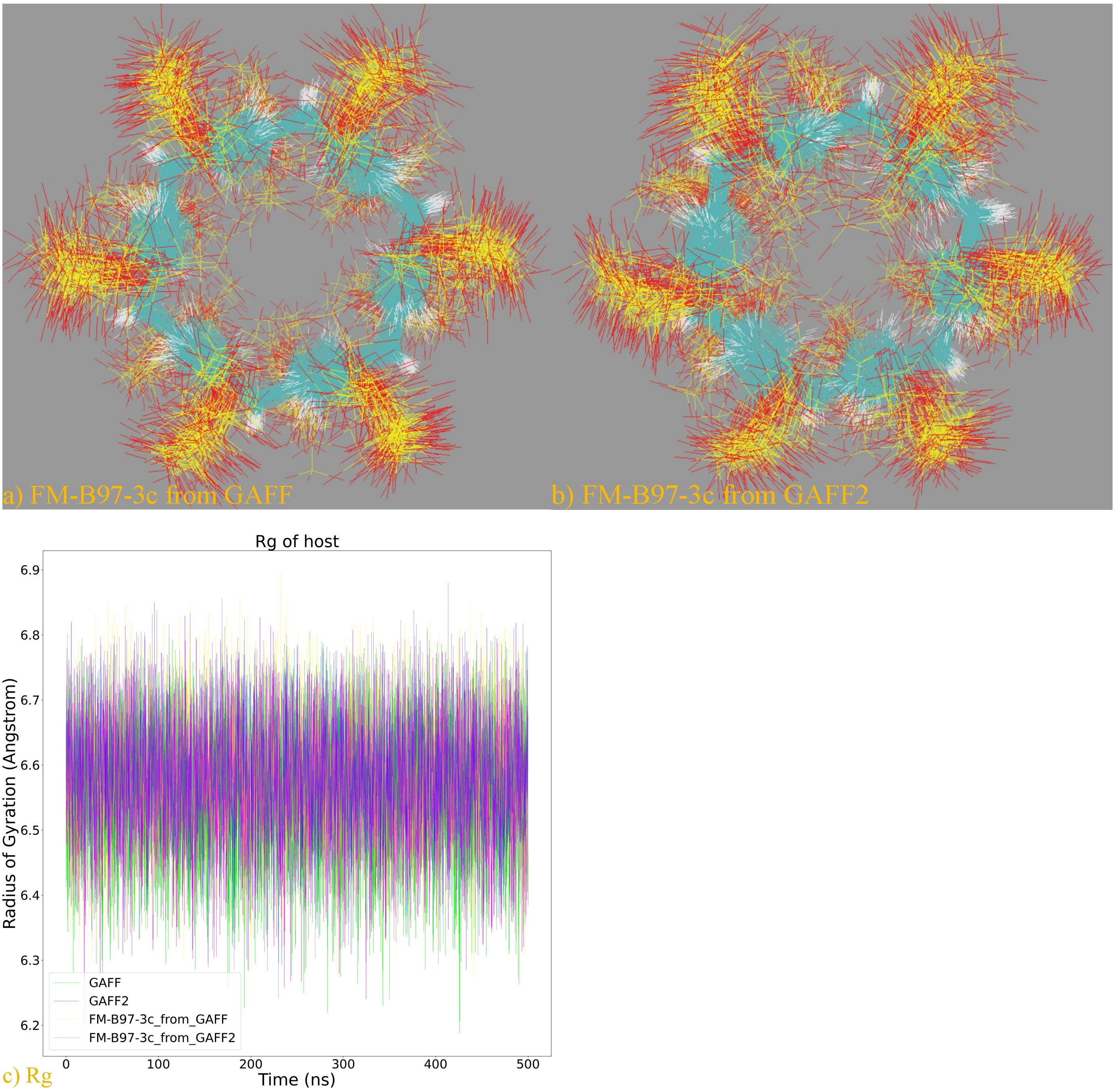

Figure S5. Deviations of force-field energetics from the B97-3c reference for the Hβ-CD host: a) the transferable GAFF and the refitted FM-B97-3c restrained to the GAFF initial guess and b) GAFF2 and the refitted FM-B97-3c-from-GAFF2 force fields. c-f) The corresponding time series of atom-specific force errors. The heavy and hydrogen atoms are numbered separately and the dividing regime is shown in the inset of the subplot a). The first 42 atoms are the carbon atoms in the CD backbone (stick type in the subplot a), the No. 43-91 atoms are the other heavy atoms (ball-type backbone oxygen and the substituting methyl groups), while the other (i.e., 92-189) atoms are hydrogen atoms. The assessment configurations and reference data independent from the fitting/training set are generated with the refitted force field FM-B97-3c for each initial guess. We can see that the force errors mainly come from heavy atoms especially the CD backbone, and hydrogen regions are satisfactorily accurate.

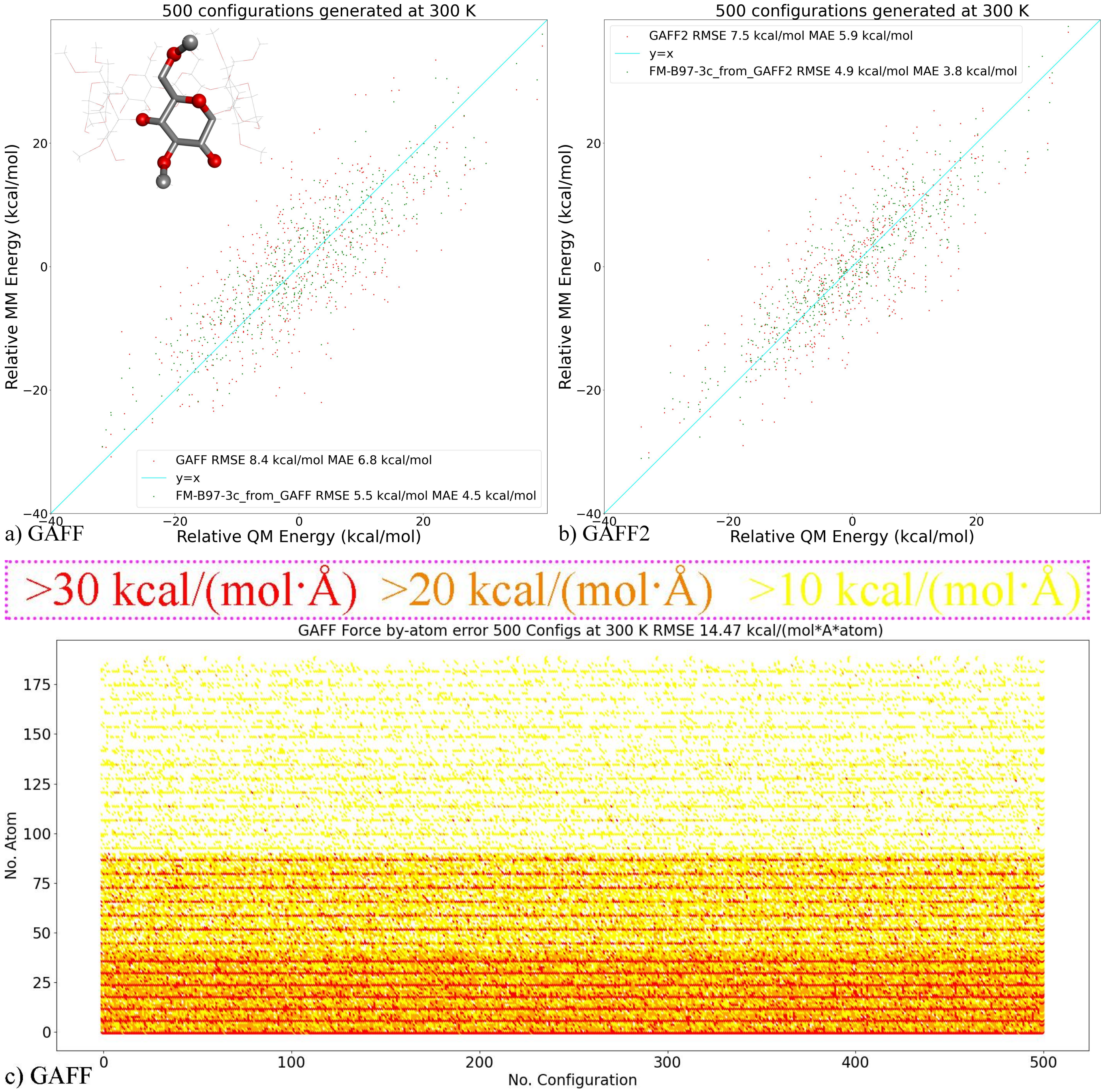

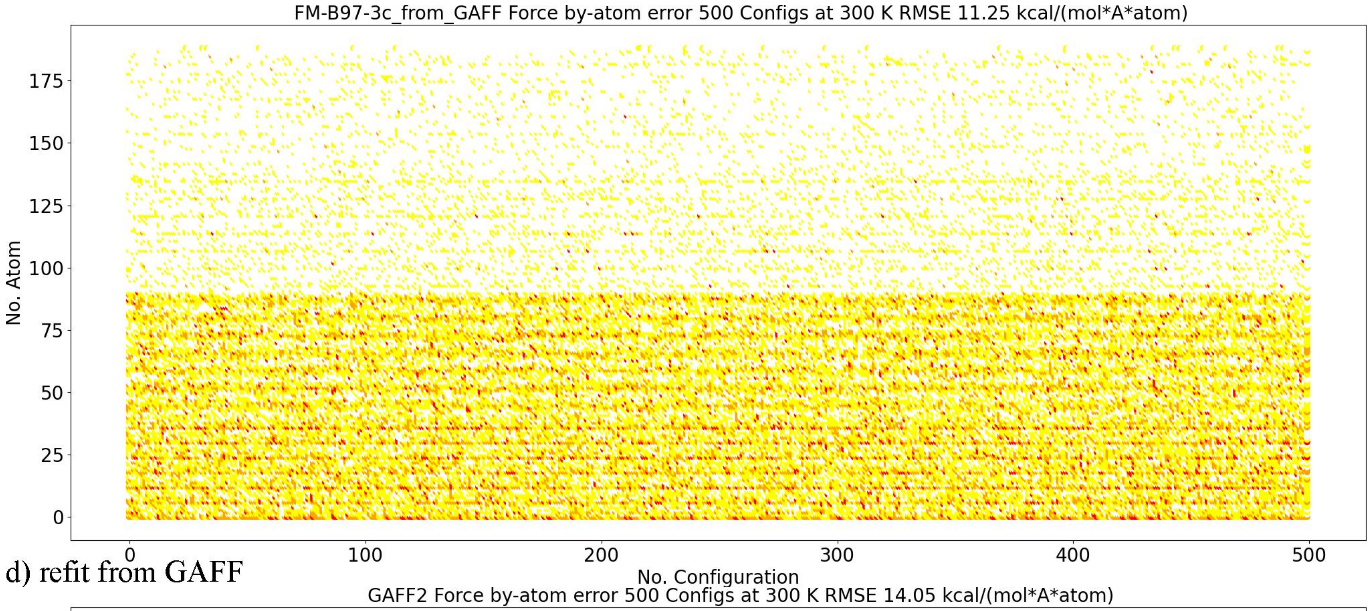

d) refit from GAFF

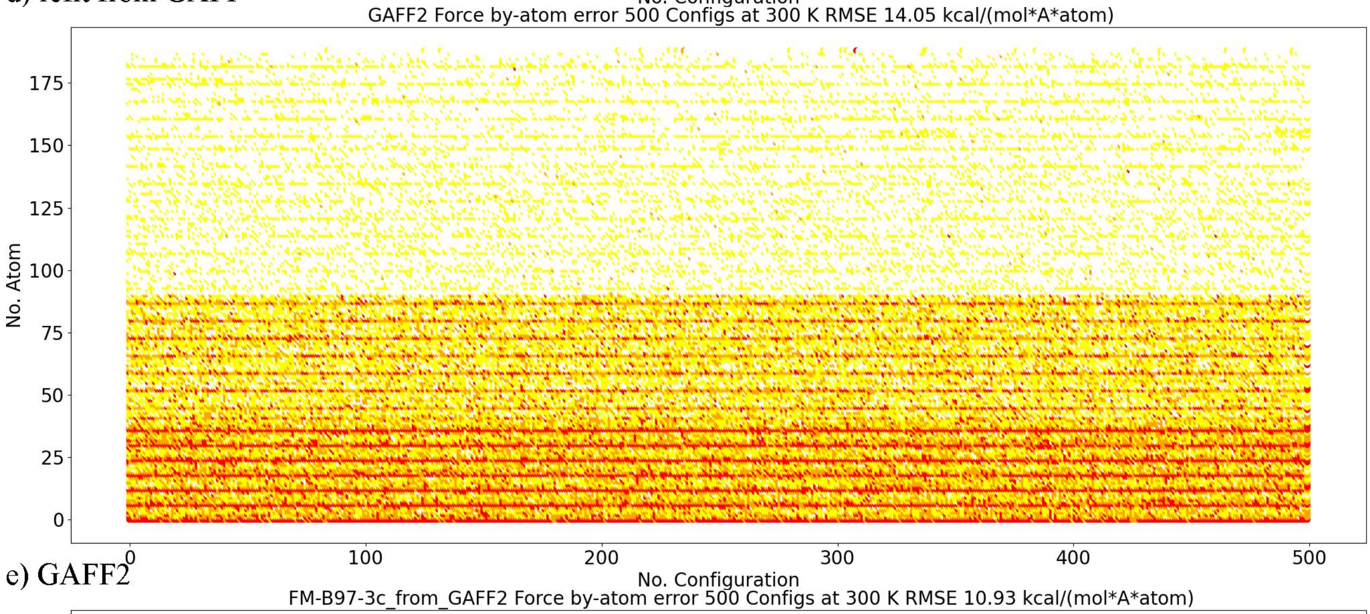

e) GAFF2

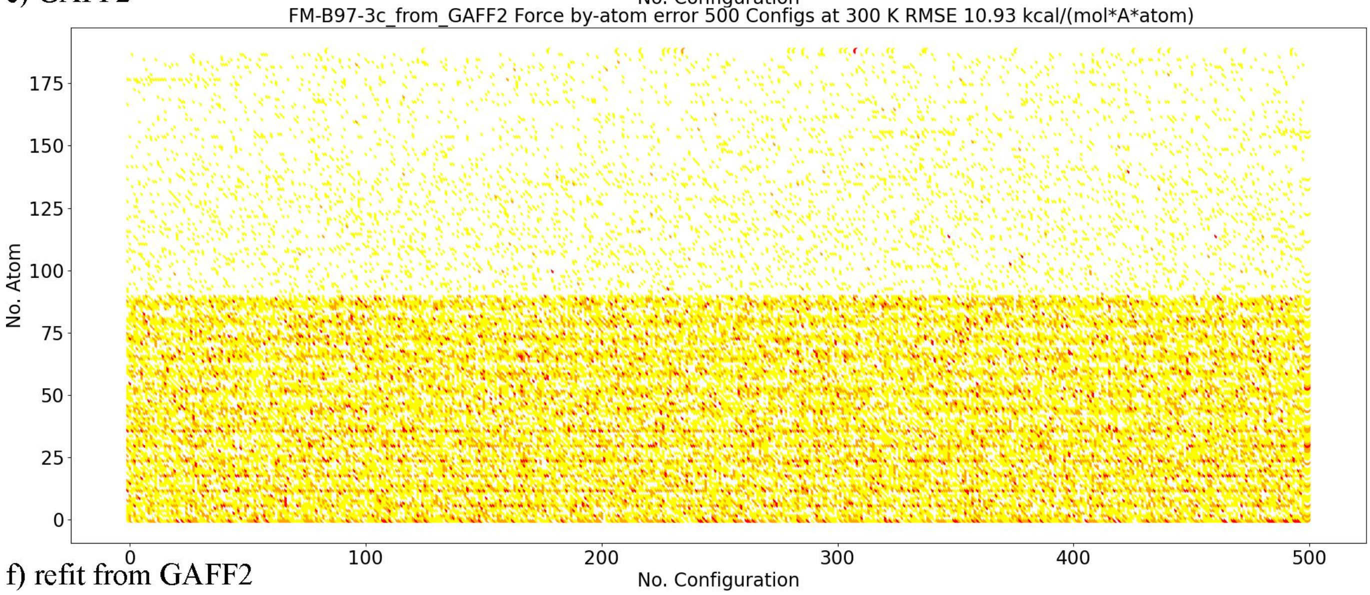

f) refit from GAFF2

Figure S6. Deviations of force-field energetics from the B97-3c reference for the OA host: a) the transferable GAFF and the refitted FM-B97-3c restrained to the GAFF initial guess and b) GAFF2 and the refitted FM-B97-3c-from-GAFF2 force fields. c-f) The corresponding time series of atom-specific force errors. The heavy and hydrogen atoms are numbered separately. The first 128 atoms are heavy atoms, while the other (i.e., 129-184) atoms are hydrogen atoms. The assessment configurations and reference data independent from the fitting/training set are generated with the refitted force field FM-B97-3c for each initial guess.

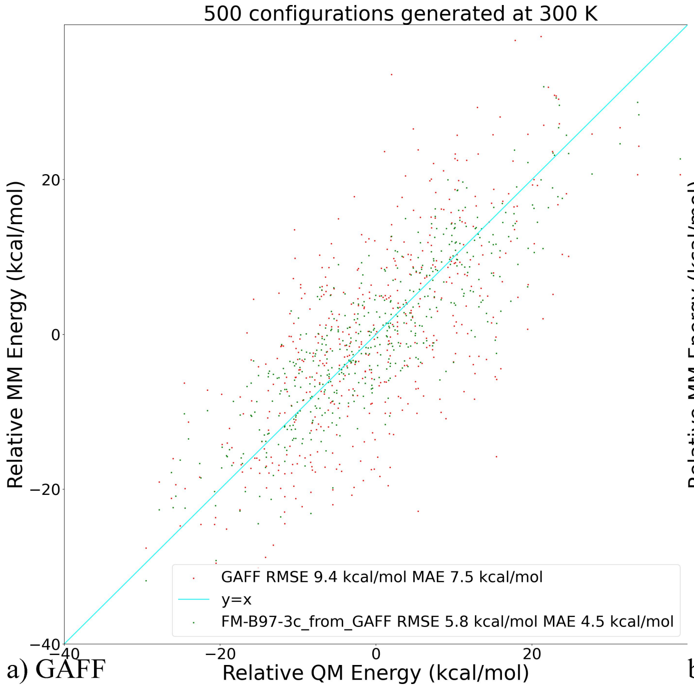

a) GAFF

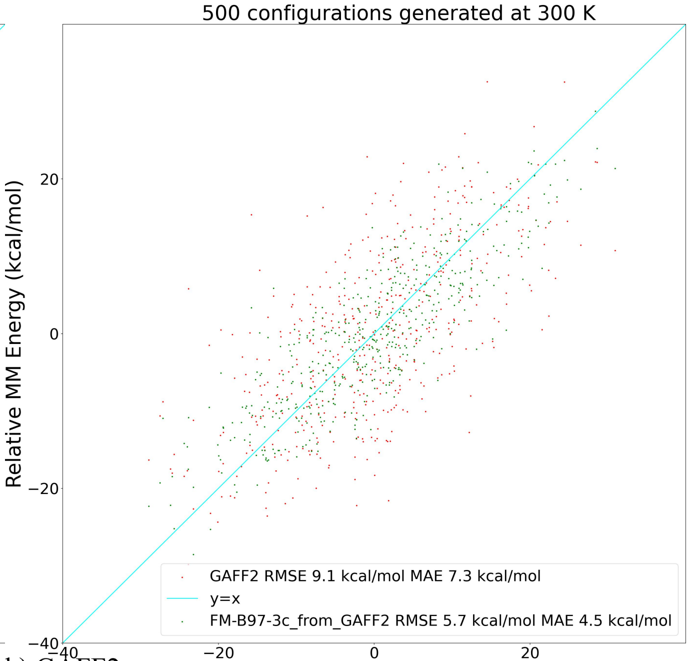

b) GAFF2

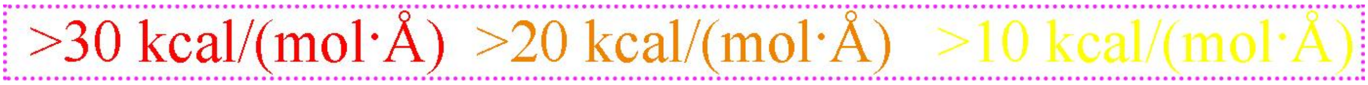

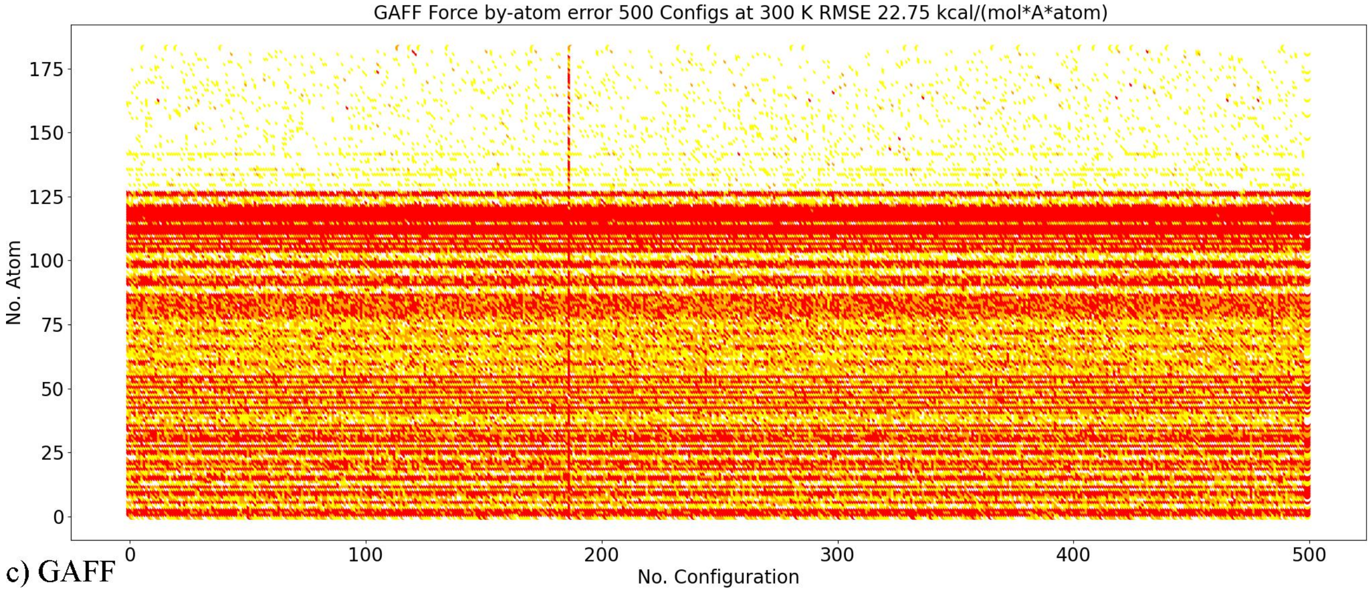

c) GAFF

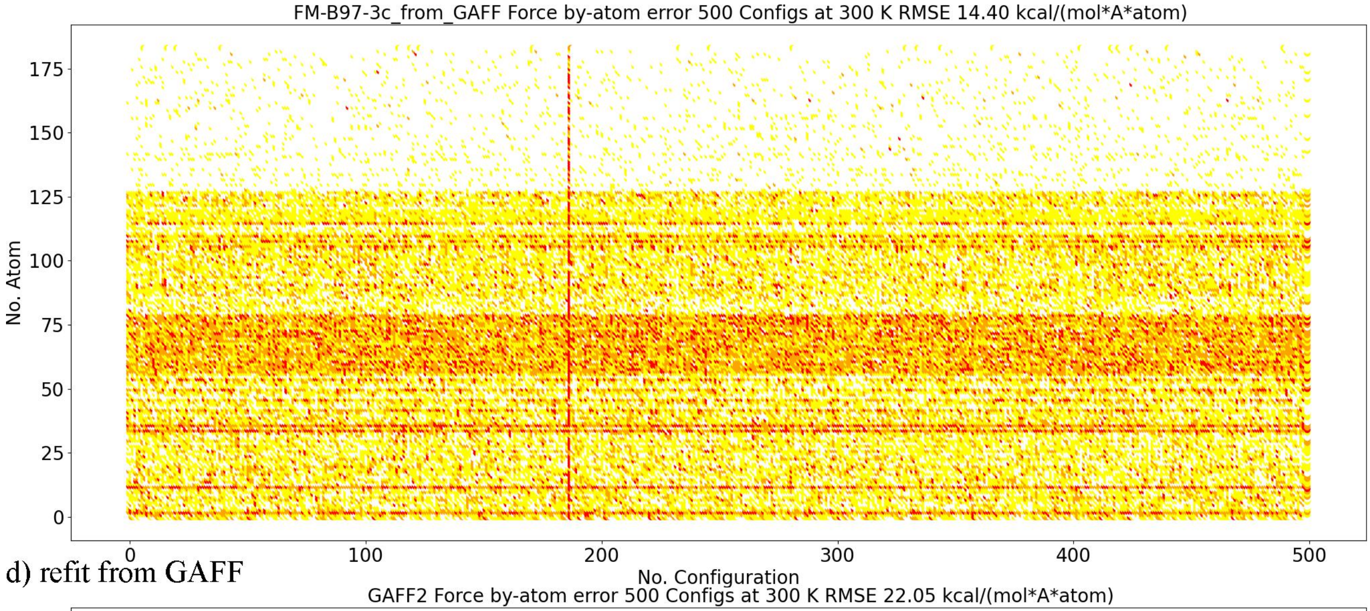

d) refit from GAFF

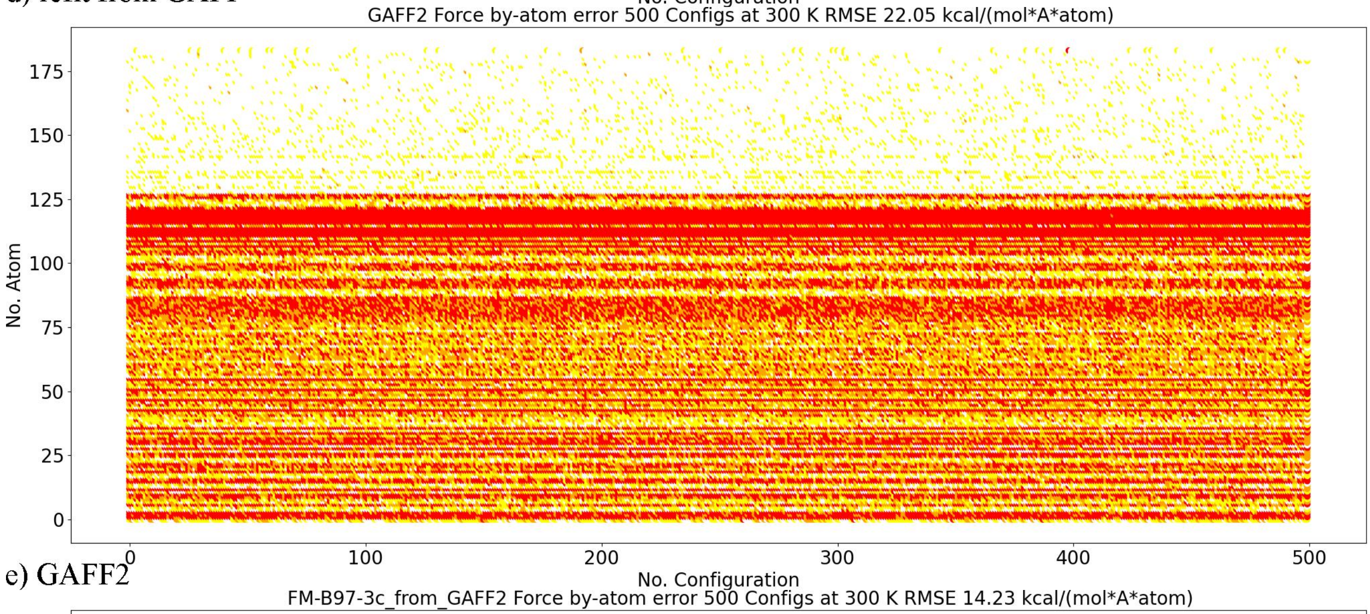

e) GAFF2

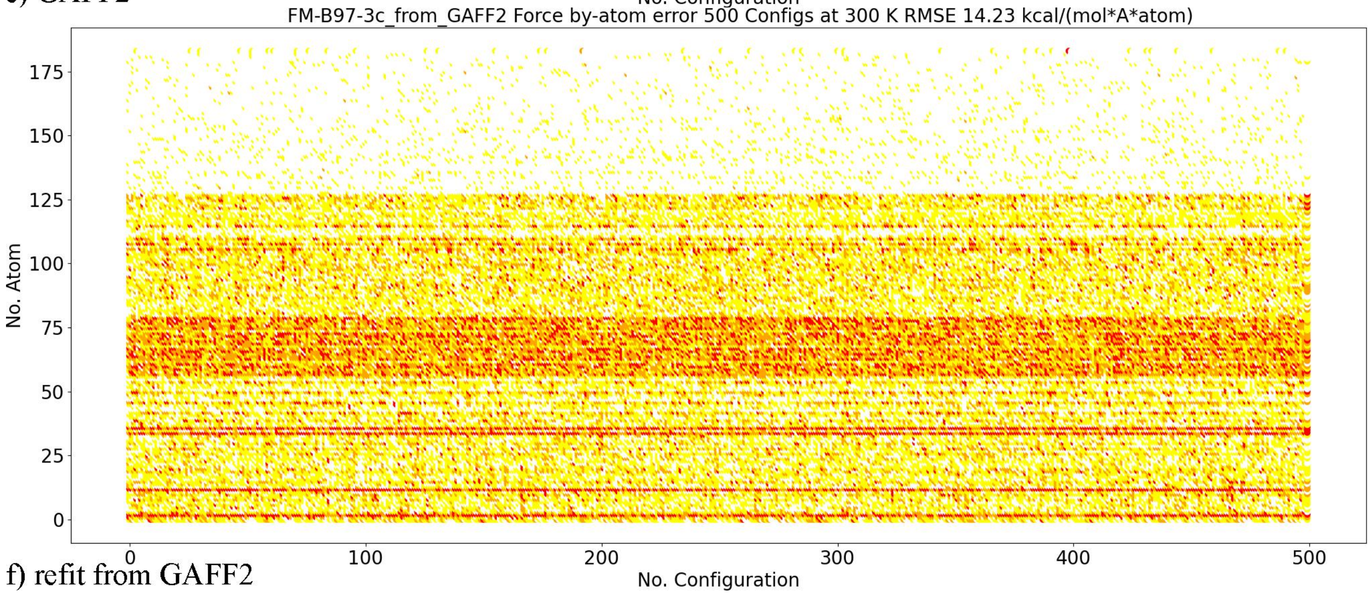

f) refit from GAFF2
